# The effects of thalidomide in a rat model of surgically-induced endometriosis

**DOI:** 10.4274/tjod.71601

**Published:** 2015-09-15

**Authors:** Murat Bakacak, Önder Ercan, Bülent Köstü, Mehmet Sühha Bostancı, Fatma İnanç, Aslı Yaylalı, Salih Serin, Ozan Balakan, Gürkan Kıran

**Affiliations:** 1 Sütçü İmam University Faculty of Medicine, Department of Obstetrics and Gynecology, Kahramanmaraş, Turkey; 2 Sakarya University Faculty of Medicine, Department of Obstetrics and Gynecology, Sakarya, Turkey; 3 Sütçü İmam University Faculty of Medicine, Department of Biochemistry, Kahramanmaraş, Turkey; 4 Sütçü İmam University Faculty of Medicine, Department of Histology and Embryology, Kahramanmaraş, Turkey; 5 Sütçü İmam University Faculty of Medicine, Department of Internal Medicine, Medical Oncology, Kahramanmaraş, Turkey

**Keywords:** animal model, Thalidomide, endometriosis, Vascular endothelial growth factor

## Abstract

**Objective:**

The aim of the study was to analyze the anti-angiogenic role of thalidomide and to assess whether thalidomide had any influence on a rat model of surgically-induced endometriosis.

**Materials and Methods:**

Endometriosis was induced through surgical induction and homologous transplantation in 16 rats. The rats were randomly separated into two groups as thalidomide (n=8) and control (n=8) groups. Using oral gavage, 100 mg/kg thalidomide 0.5 ml was administered to the first group and saline 0.5 ml to the control group. Histopathologic findings and volume analysis of implants were evaluated after 4 weeks. Vascular endothelial growth factor-A (VEGF-A) and oxidative markers were run from the fluid through peritoneal lavage.

**Results:**

The average implant volume decreased significantly in the thalidomide administrated group after treatment (53.3 and 22.9 mm^3^ respectively, p=0.012). Significant differences observed in the histopathologic scores of the thalidomide group (3 and 1 respectively, p=0.012) were not observed in the control group. Significant decreases were observed in the levels of VEGF-A and myeloperoxidase (MPO) from oxidative markers (p=0.004, p=0.037, respectively).

**Conclusion:**

Thalidomide provides volumetric and histopathologic recovery in implants particularly because the VEGF inhibition and anti-angiogenic effect, which suggests that it could be effective in the treatment of endometriosis.

## INTRODUCTION

Endometriosis is a disease seen during reproductive years that causes infertility and pelvic pain due to the settlement of endometrial tissues outside the uterine cavity. The prevalence in the general population is estimated to be approximately 10%. It is an estrogen-dependent disease that peaks between the ages of 25-34 years^(1)^. However, a rate of up to 50% has been reported from laparoscopic studies in adolescents who presented with chronic pelvic pain and dysmenorrhea^(2)^. The physiopathology of endometriosis has not yet been fully elucidated, although there are many studies and current theories in addition to the retrograde menstruation hypothesis developed by Sampson^(3)^. The basis of these hypotheses is that endometrial tissues that passes to the peritoneal cavity resist natural killer cells in direct proportion to their size. With a genetic predisposition, the endometrial tissue is implanted through transmesothelial invasion when the immune clearance mechanism and inflammatory cytokines are insufficient^(4,5)^. Neuroangiogenesis plays a crucial role in this formation, and vascular endothelial growth factor (VEGF) is held responsible initially, with mitogen and morphogen emitted from active peritoneal macrophages at the same time. It is also present in peritoneal fluid, which is correlated with the waiting phase^(6,7)^. Endometriosis formation becomes easier where the oxidant/antioxidant balance is disrupted in favour of oxidative stress and the concentration of stress markers in peritoneal fluids increases^(8,9)^.

There is a need for new medications because the hormonal therapies, anti-inflammatory agents, and complementary therapies in current use tend to simply relieve symptoms rather than healing the disease, and they have poor adverse effect profiles^(10)^.

Thalidomide is used in the treatment of many diseases because of its anti-proliferative, anti-angiogenic, and immunomodulator effects^(11)^. It demonstrates an antitumor effect particularly through the inhibition of TNF alpha, IL-8, and indirectly, nuclear factor-kB (NF-kB). These effects have also been seen in endometrial stromal cells and it has therefore been suggested that this multiple effect could be used in the treatment of endometriosis^(12)^.

For ethical reasons, the effect of thalidomide on the treatment of human endometriosis is not sufficiently known. Therefore, a surgically-induced rat model was used in this study to research the efficacy of thalidomide. The levels of angiogenic and oxidative stress markers were analyzed to show the action mechanism of thalidomide treatment, based on the hypothesis that thalidomide would cause regression of endometriotic centers through anti-angiogenic and anti-oxidative effects.

## MATERIALS AND METHODS

### Animal model and experimental design

The study included a total of 16 adult Wistar-Hannover albino nulligravida female rats, aged at least 3 months, weighing 200-250 gr and with ongoing estrous cycles. The rats were prepared for the experiment under optimum conditions (20±2 ºC, 60% humidity) in the research center with a 12-hour light/dark cycle and with standard rat food (Purina). Approval for the study was granted by Kahramanmaraş Sütçü İmam University Animal Experiments Ethics Committee. The rats were taken from the Experimental Animal Laboratory and the study was conducted in accordance with the regulations for animal experiments as determined by the National Society for Medical Research^(13)^.

Surgical endometriosis formation was confirmed with 4 weeks of a pilot scheme before the study and the adverse effects of thalidomide on the rats were observed. There after, 3 surgical phases were undertaken. All operations were performed by the same researcher in the normal manner. No rats died during the experiment.

### Surgical procedures

A 10% povidone iodine solution was used as skin antisepsis in the operations. Anesthesia was administered as 60 mg/kg ketamine hydrochloride (Ketalar Eczacıbaşı Warner-Lambert pharmaceutical industry, Levent, İstanbul) and 7 mg/kg xylazine hydrochloride (Rompun Bayer, Şişli, İstanbul) intramuscularly under aseptic conditions. The abdominal anterior wall and skin were closed using vicryl 3/0 (polyglactin 910, Medico Huaian Co., Ltd. Jiangsu, China) and silk 3/0 (Ruschmed, İstanbul, Turkey) sutures, respectively.

In the first laparotomy, endometriosis was created surgically. This was based on the method proposed by Vernon and Wilson^(14)^ and modified by Lebovic^(15)^. A midline incision approximately 4-5 cm in length was opened from 2 cm over the pubis. The uterine horns were excised from the uterotubal junctions and cervical components after ligation and were placed into into a warm and sterile phosphate-buffered saline (PBS) solution. They were then separated into 4 equal parts in such a way to include approximately 20 mm^2^ of endometrial surface and were sutured with 4-0 vicryl material to regions of intense vascularization on the intraperitoneal surface. For 2 weeks after the first surgical operation, 50 µg/kg Estradiol Depot (Jenapharm GmbH & Co, Germany) was administered intramuscularly twice a week to all rats.

The second laparotomy was conducted at the end of 2 weeks to confirm endometriosis formation and to take the necessary measurements. Endometriosis formations in 4 implanted regions for all rats were observed macroscopically at first. Based on similar studies, 3 dimensional measurements were taken of all lesions using a micrometer (length (L) x width (W) x height (H) in mm) and the spherical volume of the implants was measured using the prolate ellipsoid formula (V (mm^3^)=0.52xLxWxH)^(16)^. The lesions were excised from all the rats using a randomization table and endometriosis was also confirmed microscopically in all lesions before treatment. Histopathologic scoring was carried out according to epithelium preservation^(17)^. Implants and microscopic evaluations were photographed and recorded. Peritoneal lavage was performed with 2 mL sterile saline solution; one half was used in the evaluation of VEGF and the other was used in the evaluation of various oxidative markers. The operation was completed under sterile conditions, then the 16 rats were randomly separated into two equal groups as the treatment (thalidomide) group and the control group. A solution, prepared with distilled water to include 100 mg/kg thalidomide (Thalidomide Celgene 50 mg 28 capsules, Celgene Europe Limited, UK), was administered to the treatment group through 0.5 ml oral gavage for 2 weeks and isotonic solution was given to the control group in the same way^(17)^.

All endometriotic implants were excised in the third surgical phase and spherical volume calculation, histopathologic scoring, and peritoneal lavage for the examined markers were performed. The rats were sacrificed by decapitation under anesthesia following the completion of the surgical phases.

### Histopathologic analysis

**Figure 1:**
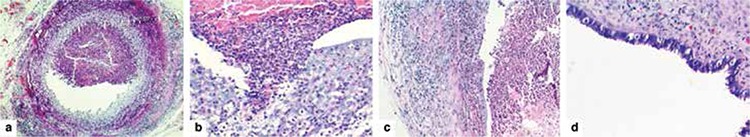
Examination of the sections after biopsy and histopathologic scoring of the materials stained with hematoxylin and eosin. a) Lack of epithelium in sections; score 0 (x40 enlargement), b) Lightly preserved epithelium layer; score 1 (x200 enlargement), c) Reasonably preserved epithelium layer; score 2 (x100 enlargement), d) Well preserved epithelium layer; score 3 (x200 enlargement)

The materials were then embedded in paraffin blocks and 5-mm-thick sections were taken using a microtome (Leica RM2145) and stained with hematoxylin and eosin. The preparations were examined under an optical microscope and the endometrial implant samples were scored based on the preserved epithelium cell. The classifications were as follows; 0=no epithelium; 1=lightly preserved epithelium layer; 2=reasonably preserved epithelium layer with leukocyte infiltration; 3=well preserved epithelial layer (Figure 1)^(17)^.

### Evaluation of oxidative markers and vascular endothelial growth factor-A

Superoxide dismutase (SOD), glutathione peroxidase (GPx), and catalase (CAT) were analysed as antioxidants^(18,19)^. In the peritoneal fluid samples taken during the surgical intervention, nitric oxide (NO)^(20)^ from reactive oxygen radicals; lipid peroxidation product malondialdehyde (MDA); and myeloperoxidase (MPO), which is mainly present in acute inflammation, were examined using a Shimadzu UV Spectrophotometer UV-1800 (Japan) before and after the treatment^(20,21,22)^. In addition, the measurement of VEGF-A, which is one of the important regulator peptides defined in angiogenesis, was examined using a Multiscan FC Microplate Photometer (Thermo Scientific, Finland) device using Rat ELISA (Wuhan EIAAB Science Co., LTD. Wuhan, China) kit^(23)^.

### Statistical analysis

The Statistical Package for the Social Sciences 21 (SPSS, Chicago, IL, USA) and PAST programs were used in the analysis. The compatibility of univariate data to normal distribution was analyzed through the Kolmogorov-Smirnov test, Shapiro-Wilk test and Coefficients of Variation and the same analysis was carried out with the Mardia, Doornik and Omnibus test for multivariate data. While Bootstrapping was applied for the independent-Samples t-test, the Monte Carlo simulation technique was used for the Mann-Whitney U test in the comparison of 2 independent groups. Bootstrapping was applied for the paired-Samples t-test, and the Monte Carlo simulation technique for the Wilcoxon Signed Rank test for the two repeated measurements of dependent variables. Bootstrapping was applied for the general linear model-repeated Anova test to analyze the interaction of the repeated measurements of variables according to groups. Quantitative data was expressed as mean ± standard deviation (SD) and median ± interquartile range (IQR) values in the tables. Categorical data were stated as number (n) and percentages (%). The data were analysed at 95% confidence level, and a value of p<0.05 was accepted as statistically significant.

## RESULTS

**Figure 2:**
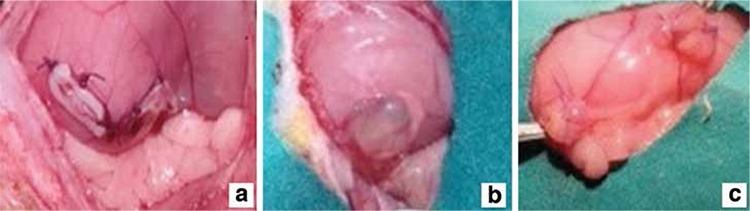
a) Induction of endometriosis at first laparotomy, b) Visualisation of cystic, viable endometriotic implant on second surgery,c) Smaller endometriotic implants after thalidomide treatment on the last surgery

All rats were observed daily throughout the study. No complications were seen during the surgical procedures or postoperative follow-up period. No adverse effects were observed either from the anesthesia medication or the thalidomide treatment. All rats survived to the end of the study. Endometriosis formations were observed in all the rats when the lesions were removed and analyzed in the second surgical intervention after induction, and the lesions were morphologically similar (Figure 2).

**Figure 3:**
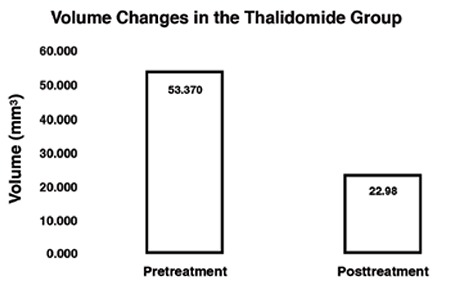
Levels of change in the implant volume before and after the treatment in the thalidomide group

**Figure 4:**
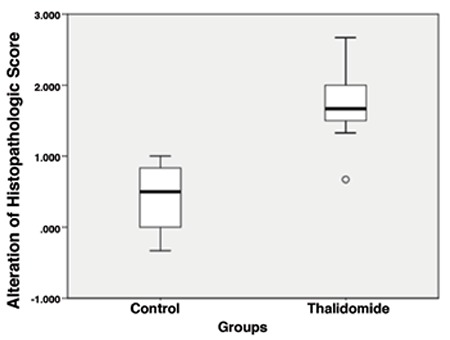
Examination of histopathologic score changes before and after the treatment in both groups

In the thalidomide group, the post-treatment mean implant volume (22.9±22.2 mm^3^) was observed to have decreased significantly compared with the pre-treatment mean implant volume (53.3±33.1 mm^3^) (p=0.012). In the control group, although a total increase was observed in the mean values, no significant change (102.49±87.70, 112.8±112.2, mm^3^ respectively, p=0.738) was determined (Table 1, Figure 3). When both groups were analyzed in respect of histopathologic score, the post-treatment score (1±0.83) regressed significantly compared with pre-treatment (3±1) in the thalidomide group, whereas there was no significant regression in the control group (3±0.5, 2.33±0.67, respectively, p=0.096) (Table 1, Figure 4).

**Figure 5:**
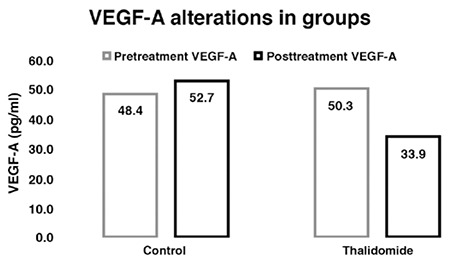
Levels of vascular endothelial growth factor-A changes before and after the treatment in both groups

From the analyzed oxidative markers, only MPO decreased significantly after thalidomide treatment (34.56±27.80, 10.33±4.09, U/lt. respectively, p=0.037). No significant change was seen in the control group. No significant change was observed in the treatment group in terms of NO and other markers (Table 2). The levels of VEGF-A, which is an important neuro-angiogenic, decreased significantly in the treatment group (p=0.005); the increase in the control group also demonstrates the significant difference between the two groups (p=0.004) (Table 2, Figure 5).

## DISCUSSION

Although various approaches to endometriosis treatment have been suggested by physicians in recent years, there is as yet no ideal treatment. Therefore, by considering the physiopathology and progression of the disease, thalidomide was selected for analysis in this study on a surgically-induced rat endometriosis model. The effects of heterologous transplantation on immune response were excluded by the use of the homologous transplantation method in the model^(14)^.

A complex interaction of immune response, neuroangiogenesis, and oxidative balance is thought to be responsible for the formation and progression of endometriosis^(6,9)^. Thalidomide may have the response to these questions of balance and interaction. It is used primarily in dermatologic and inflammatory diseases, and in cancers such as multiple myeloma. It has been reported to provide anti-proliferative effects with NF-kB inhibition, anti-inflammatory effect with IL-8, and TNF alpha inhibition, also an anti-angiogenic effect with VEGF inhibition, and an antioxidant effect, particularly with NO inhibition^(11,12)^.

In a study by Scarpellini et al.^(24)^ 300 mg/kg thalidomide was added to the therapy of 10 women with endometriosis, in whom relapse had been observed after previous GnRH treatment. Remission in symptoms was reported in 8 of the 10 patients. Even though the number of patients was small, no relapse in long-term follow-up was noteworthy in this study.

In the current study, significant decreases were observed in the implant volume and histopathologic scores of the treatment group, which were not seen in the control group, thereby supporting the hypothesis that thalidomide would downgrade the disease. This is a similar result to that reported by Azimirad et al.,^(25)^ who first analyzed the effects of thalidomide on endometriosis histopathology in rats. From that study, significant decreases in post-treatment histopathologic scores and statistically significant differences in VEGF, IL-6, and leukocyte counts were observed between the treatment and control groups. In the current study, VEGF values were also seen to have regressed to a statistically considerable extent in the thalidomide group after ELISA measurements. The decrease in VEGF values can be thought to be as important as histopathologic and volumetric regression. Mclaren et al.^(6)^ first reported that VEGF peritoneal fluid concentrations showed cyclic oscillation and that there was a significantly higher level in cases of endometriosis. It was also stated that the passage to the proliferative phase in this cyclic oscillation was different in the endometriosis group compared with a healthy control group, and implantation and proliferation were formed with this high level of cyclic oscillation. Shifren et al.^(7)^ also reported VEGF secretion 46% higher than normal level in an estradiol-administrated group in a study in which VEGF mRNA levels were analyzed in vivo in human endometrial cells; it was reported that VEGF could be an estrogen-dependent angiogenic factor.

There are any previous reports in the literature on endometriosis etiopathology with research on the effects of oxidative stress on the growth and adhesion formation of endometrial implants in the peritoneal cavity^(8,9)^. In the comparison of before and after treatment in the thalidomide group, significant decreases were seen in MPO enzyme levels, which are known to increase, particularly in acute inflammation. No significant increase was observed in SOD, GPx, and CAT enzymes, which are known as antioxidants. While the reasons for this may be that the oxidative stress that causes endometriosis could be chronic, the experiment time was too short to understand the antioxidant effect of thalidomide and measurements could not be confirmed with ELISA kits. It could also be interpreted that the antioxidant effect of thalidomide was not followed in the constituted model. That the effect is shown with NO inhibition has been particularly emphasized in the literature; therefore, the fact that medication did not undergo a significant change in the current study was consistent with this interpretation^(11,12)^. Our suggestion in this particular area is that researchers should approach the subjects of marker preference, working time, and device selection. Moreover, it should be kept in mind that surgery may also disrupt this balance depending upon oxidative stress and acute inflammation.

The main limitation of this study is that immunohistochemical evaluation was not performed. The shortness of the experiment duration also constituted a restriction with regards to the long-term effects of treatment and its analysis with a 4^th^ surgical procedure related to relapse formation. Again, a longer experiment duration would be helpful for the analysis of oxidative balance together with the progress of chronic inflammation.

In conclusion, thalidomide, which has previously been known for its teratogenous effect, has recently become prominent in the treatment of many serious diseases. The outcomes of this study report positive results in the sense of the regression of lesions and repressing angiogenesis; thalidomide could be used in the treatment of endometriosis. Future studies with more experimental subjects are required to support the combined and separate use of thalidomide with regard to long-term results and adverse effects.

## Figures and Tables

**Table 1:**
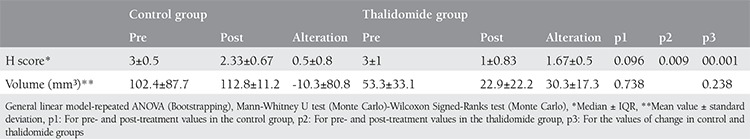
Comparison of calculated spherical volumes and histopathologic scores of endometriotic implants before and after the treatment

**Table 2:**
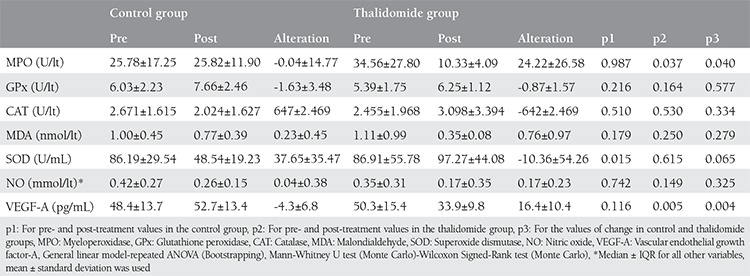
Examination of pre- and post- treatment values of oxidative marker and vascular endothelial growth factor-A levels received from the peritoneal washing materials of the rats
